# Dietary intake, obesity, and metabolic risk factors among children and adolescents in the SEACO-CH20 cross-sectional study

**DOI:** 10.1038/s41598-024-61090-7

**Published:** 2024-05-17

**Authors:** Amutha Ramadas, Hussein Rizal, Sutha Rajakumar, Jeevitha Mariapun, Mohamed Shajahan Yasin, Miranda E. G. Armstrong, Tin Tin Su

**Affiliations:** 1 Jeffrey Cheah School of Medicine and Health Sciences, Monash University Malaysia, 47500 Bandar Sunway, Malaysia; 2 South East Asia Community Observatory (SEACO), Jeffrey Cheah School of Medicine and Health Sciences, Monash University Malaysia, 47500 Bandar Sunway, Malaysia; 3 Clinical School Johor Bahru, Jeffrey Cheah School of Medicine and Health Sciences, Monash University Malaysia, 80100 Johor Bahru, Malaysia; 4 Centre for Exercise, Nutrition & Health Sciences, School for Policy Studies, University of Bristol, Bristol, BS8 1TZ UK

**Keywords:** Diseases, Endocrinology, Risk factors

## Abstract

We investigated the association between dietary intake and metabolic risk factors in children and adolescents within a semi-rural Malaysian community. Using an interviewer-led questionnaire, we surveyed 623 participants aged 7–18 from the South East Asia Community Observatory (SEACO). Anthropometric and blood pressure data were collected from all participants, while a subset (n = 162) provided blood samples for biomarker analysis, including fasting blood glucose (FBG), total cholesterol (TC), triglycerides (TG), high-density lipoprotein cholesterol (HDL-C), and low-density lipoprotein cholesterol (LDL-C). Metabolic syndrome was determined using the International Diabetes Federation’s Definition of Metabolic Syndrome in Children and Adolescents. Most participants were Malay (66.8%), with a median household income of MYR1,500 and a balanced sex distribution. Cereals, processed foods, beverages, fruits, and vegetables were commonly consumed. Obesity and abdominal obesity were prevalent, affecting more than a third of participants. Adherence to dietary recommendations was generally poor (ranging from 19.9 to 58.1%) and varied across age, sex, and ethnicity. Notably, some food groups displayed unexpected associations with health markers; for instance, fruit consumption was linked to abdominal obesity in children (abdominal obesity vs. normal: 2.4 servings/day vs. 1.6 servings/day). These findings emphasise the necessity of longitudinal studies to explore the complex relationship between diet and long-term health outcomes, including cardiometabolic diseases, while acknowledging the unique challenges posed by the COVID-19 pandemic on data collection and analysis.

## Introduction

Limited diversity and inadequate consumption of essential food groups, such as fruits and vegetables, often characterise the dietary intake of children and adolescents. Overemphasis on plant-based foods in their diets, alongside a concerning trend of increasing consumption of high-energy snacks and beverages, has been highlighted in the literature^1^. This pattern contributes to a dual burden of malnutrition, with both undernutrition and overnutrition prevalent among this population. Mates et. al.^2^ echoed this in their review, identifying urban areas and boys as particularly vulnerable groups with poorer dietary patterns.

Poor diet quality is a common concern, with declining dietary standards observed during the transition from childhood to adolescence^3^. This trend is a major contributor to the rising incidence of non-communicable diseases. Longitudinal studies emphasize the importance of addressing dietary patterns early in life, as childhood dietary behaviours tend to track into adulthood. For instance, Craigie and colleagues^3^ have provided compelling evidence supporting this phenomenon. Their research highlights the importance of early intervention to address dietary patterns and habits during childhood and adolescence. By targeting these formative years, interventions have the potential to positively influence long-term health outcomes, mitigating the risk of chronic diseases later in life. This underscores the critical role of early dietary interventions in promoting lifelong health and well-being.

Appanah et al.^4^ further highlight the association between poor diet quality and adverse health outcomes among Malaysian adolescents, including cardiometabolic risk factors. However, despite the growing awareness of the importance of good nutrition during childhood and adolescence, there remains a lack of comprehensive studies exploring the impact of diet quality on clinical outcomes in these age groups, particularly in regions such as Malaysia^5^. The existing evidence underscores the critical role of good dietary intake in promoting the health and well-being of children and adolescents, emphasizing the need for targeted interventions and policies to address dietary inadequacies and improve long-term health outcomes in this population.

Metabolic syndrome (MetS) is an emerging area of research among children and adolescents. MetS is characterised by the clustering of risk factors including abdominal obesity, elevated blood pressure (BP), fasting blood glucose (FBG) and triglyceride (TG), and low high-density lipoprotein cholesterol (HDL-C), which increases the risk of developing type 2 diabetes (T2D) and cardiovascular diseases (CVD) later in life^6^. While the assessment of paediatric MetS is still debatable^7^, existing data suggest approximately 3% of children and 5% of adolescents have MetS, with some variation across nations and regions^8^. This translates to more than 25 million children aged 6–12 years and more than 35 million adolescents aged 13–18 years suffering from MetS. The prevalence varies significantly by age, ethnicity and location^9^. This is an emerging public health issue in low and middle-income countries (LMICs) as a recent systematic review reported MetS was found in 4.0% (IDF)^10^, 6.7% (ATP III)^11^ and 8.9% (de Ferranti)^12^ of children and adolescents in these nations^13^.

Although a recent Malaysian community-based study reported a lower MetS prevalence of 4.8% in children^14^, Wan Mahmud et al.^15^ reported a high MetS prevalence of 56% among children with obesity and adolescents among those referred to obesity clinics in tertiary care. The study suggests children with MetS were 14 times more likely to be severely obese. Furthermore, these children exhibited higher odds of having increased FBG, TG and low HDL-C. Earlier studies also reported significantly poorer biochemical profiles, higher body fat percentages and anthropometric measures in overweight and obese children^16,17^. Readily available access to calorie-dense, nutrient-poor foods and a lack of physical activity have contributed to a rapid rise in the prevalence of paediatric obesity, which is the primary risk factor for paediatric MetS^15,18,19^.

Paediatric MetS is strongly associated with T2D and CVD^20,21^. The chronic nature of MetS emphasizes the importance of identifying risk factors specific to the population that can be addressed later. Together with the importance of physical activity and adopting a healthier lifestyle, understanding the dietary intake of the community is an essential part of reducing the risk of paediatric MetS and its progression to adulthood complications.

Mohammadi and colleagues (2019)^22^ reviewed the association between dietary patterns, physical activity, and metabolic risk factors among Malaysian adolescents. Their research revealed that obese and overweight adolescents exhibited distinct dietary behaviours compared to their normal-weight counterparts, including higher consumption of energy and macronutrients, as well as a greater tendency to skip meals. However, despite these findings, the review highlighted a notable gap in the existing research literature regarding the relationship between dietary habits and other metabolic risk factors such as lipid profile and BP^22^. This gap suggests a need for further investigation into the comprehensive effects of dietary patterns on various metabolic parameters among adolescents, particularly in the Malaysian context. Additional research in this area could provide valuable insights for developing targeted interventions aimed at mitigating metabolic risks and promoting better health outcomes among adolescents.

Hence, we aim to explore the association between dietary intake, obesity and metabolic risk factors among children and adolescents in a semi-rural Malaysian setting.

## Methods

### Study design and participants

South East Asia Community Observatory (SEACO) is a dynamic community observatory cohort of 13,335 households that have been surveyed since 2012 in Segamat, a semi-rural region in the state of Johor Darul Takzim, Malaysia. The assessments conducted in this cohort include questionnaire surveys, blood tests, and physical measurements^23^. The SEACO Health Round Survey 2018 (HR-2018) occurred between July 2018 and August 2019. Subsequently, the SEACO Child Health 2020 (SEACO-CH20) study was conducted to obtain device-measured physical activity and diet measures among Malaysian children and adolescents (aged 7–18) in a subsample of the main SEACO cohort.

The inclusion criteria were children and adolescents aged 7–18 from the SEACO cohort. The participants were sampled from 3 out of the 5 sub-districts of the SEACO cohort—Jabi, Sungai Segamat and Gemereh. Participants were excluded if their parents could not give assent during data collection due to work commitments. Participant information sheets were distributed, and informed consent was taken from parents/guardians on behalf of their child.

The minimum sample size required for this study was determined using Raosoft software^24^ with a response distribution of 50% from a population size of 38,712 and a margin of error of 5% with a confidence level of 95%. The recommended sample size is 381 children and adolescents.

**Figure 1:**
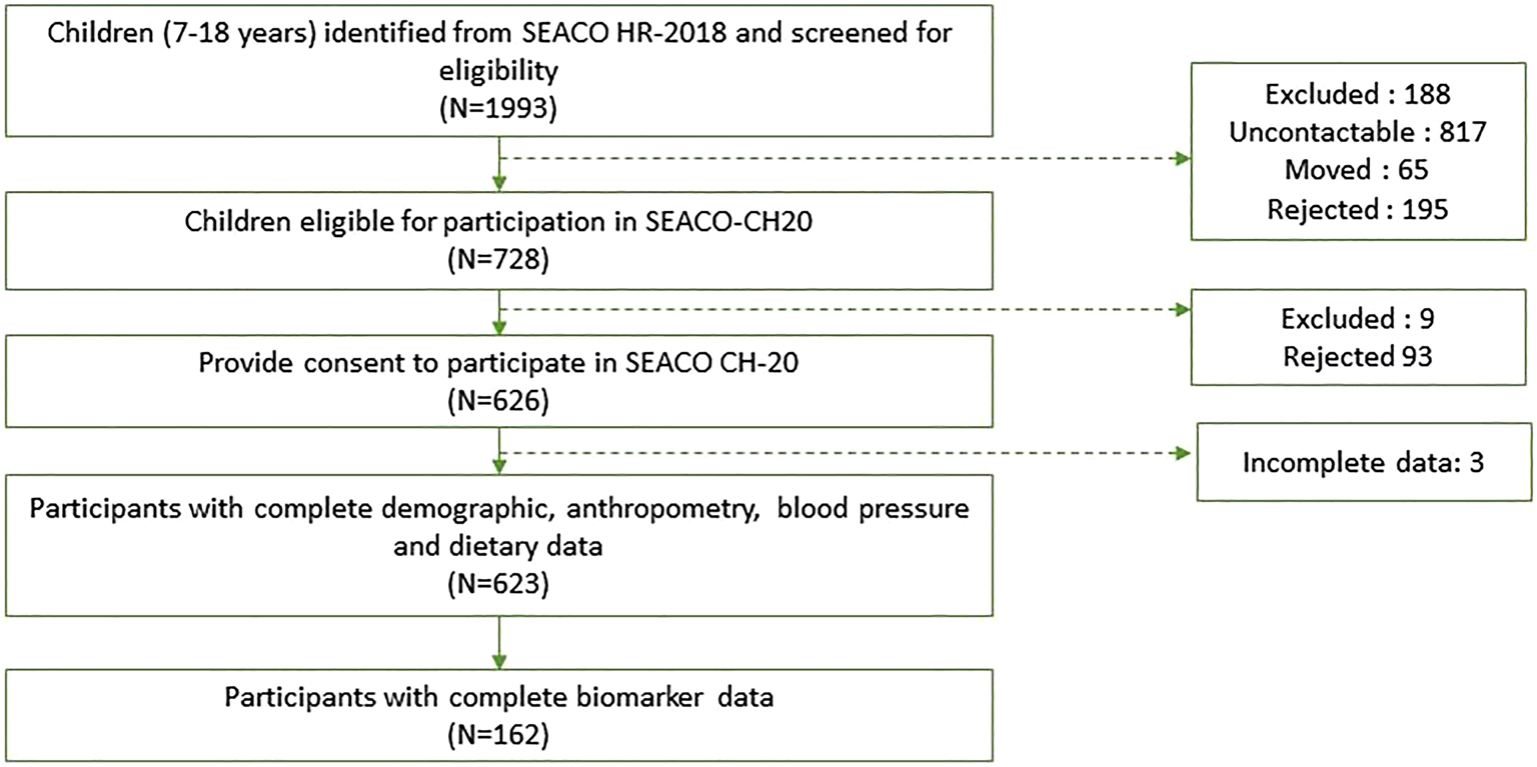
Study flow chart.

Parents of the 728 eligible participants consented to allow their children to participate in the study. Out of these, 623 participants completed the questionnaire, and a subset of 162 provided blood samples (Fig. 1). Trained fieldworkers conducted a face-to-face interview from October 2021 to July 2022, during which the participants self-administered the questionnaire. Ethical approval for SEACO-CH20 was obtained from the Monash University Human Research Ethics Committee on 17/03/2020 (Project ID: 23271) prior to data collection. The study was conducted in accordance with the Declaration of Helsinki for experiments involving humans.

### Measures

#### Demographic characteristics

The research team extracted information about children’s age, sex, and ethnicity from the SEACO HR 2018 data set. The participants were divided into two age groups: childhood (7–12 years) and adolescence (13–18 years), corresponding to primary and secondary school ages in Malaysia. The study also reported the ethnicity of the children as Malay, Chinese, or Indian. Additionally, the survey reported household income in Ringgit Malaysia (MYR).

#### Dietary intake

A short self-administered semi-quantitative food frequency questionnaire (SFFQ), adapted from the Malaysian Adults Nutrition Survey (MANS) FFQ^25^ was used to collect information on dietary intake. The SFFQ consisted of 45 food items listed in eight food groups. A list of food items according to food groups is presented in Supplementary Table S1. The SFFQ was administered during the house visits. The participants were asked about the frequency of intake of each food item (per day, week or month), and the number of servings consumed each time they ate a particular food item. Photographs depicting sample serving sizes were also included in the SFFQ to aid with the estimation of serving size. Each food item listed was given a standard serving size based on the Malaysian Dietary Guidelines 2020^26^. Dietary intake of specific food items was estimated based on the following formula: number of servings of food A = (number of servings of food A) × (frequency of intake of food A per day). Subsequently, the number of servings of food items in a particular food group was totalled to give the total number of servings per food group per day. The number of servings is also compared to the recommended servings for the participant’s age and sex according to the Malaysian Dietary Guidelines for Children and Adolescents 2013^27^.

#### Anthropometric and physical measures

Data collectors measured height and weight using a Transtek digital weighing scale and height gauge and body mass index (BMI) was calculated and converted to age-adjusted standardised z-scores using the WHO 2007 BMI reference for children aged 5–19 (BMI z-score)^28^. Children were classified as underweight, overweight and obese according to the definitions outlined by the WHO^28,29^, if the standardised BMI z-score was <  − 2, >  + 1 and <  = 2, and >  + 2 standard deviations from the mean, respectively, with the remaining children classified as a healthy weight. Waist circumference (WC) was measured after determining the midpoint between the last rib and the upper edge of the iliac crest on the right-hand side. The WC measurement was measured using Myotape Body Tape Measure (Accufitness, Denver United States). Systolic and diastolic BP were measured three times using the Digital Blood Pressure Monitor (HEM-907) with a 5-min interval between measurements, with the patient having remained seated for more than 5 min. The arterial pressure value was determined from the average of the last two measurements^30^. Both WC and BP were measured by participants’ family members to avoid close contact with data collectors and participants. The research team prepared brochures and videos guiding the family members on measuring WC and BP, and measurements were closely monitored and supervised by the trained data collectors.

### Blood sample collection

The blood sample was collected by a privately managed Ministry of Health-certified local laboratory within the study location every Friday and Saturday morning, in accordance with the schedule. Respondents were required to fast from 10 pm on Thursday until the following morning (Friday). Hence, the blood samples were collected from the participants in a fast state and verified by data collectors before being taken. Trained phlebotomists drew intravenous blood samples while using suitable personal protective equipment. The same private laboratory analysed FBG and blood lipid profiles such as total cholesterol (TC), TG, HDL-C and low-density lipoprotein cholesterol (LDL-C). The quality of the samples was maintained via several steps, including visual inspection to detect abnormalities, minimum quantity, proper sealing and absence of leakage or contamination, as well as accurate labeling.

### Definition of metabolic syndrome

The five metabolic risk factors (WC, BP, FBG, TG and HDL-C) were screened in the study participants. A total of 162 participants were assessed for the following criteria per the IDF Definition of Metabolic Syndrome in Children and Adolescents by the presence of WC ≥ 90th percentile along with two or more of the following four criteria: FBG ≥ 100 mg/dL; TG ≥ 150 mg/dL; HDL-C ≤ 40 mg/dL; systolic BP ≥ 130 mmHg and/ or diastolic BP ≥ 85 mmHg^31^.

### Statistical analysis

Statistical analyses were performed with IBM SPSS Statistics for Windows, Version 28.0 (Armonk, NY: IBM Corp). Normality was assessed using visual inspection of the histogram and normality plots. Continuous variables with normal distribution (age, blood pressure, anthropometry, blood biomarkers) were presented as mean and standard deviation (SD), while variables with skewed distribution (daily servings of food intake) were presented as median and interquartile range (IQR). Categorical variables are presented as frequencies and percentages. The Mann–Whitney U or Kruskal–Wallis tests were used to analyse the difference in food intake between demography and metabolic indicators. Pairwise comparisons with Bonferroni corrections were performed if the Kruskal–Wallis test resulted in any significant differences in median servings of food intake. The Chi-square test was performed to assess the association between adherence to guidelines and demographic characteristics. Sub-group analyses were performed by comparing median food intakes between metabolic indicators according to demographic groups. The level of significance was set at *p* < 0.05.

## Results

### Characteristics of the study participants

The study included 623 participants, primarily children aged 7–12 and adolescents aged 13–18. Table 1 provides descriptions of overall study participants and according to age groups. The mean age of the study participants was 12.7 + 2.8 years. The majority of participants were Malay, comprising 66.8% of the total. Additionally, there was a nearly equal distribution based on sex, with males accounting for 51% and females for 49%. The median household income was MYR1,500. Demographic characteristics were similar between the age groups.

**Table 1:** Description of the study population (N = 623).

			Total (N = 623)	Children (7–12 years) (n = 285)	Adolescents (13–18 years) (n = 339)
Demography	Age (years)	Mean (SD)	12.7 (2.8)	10.1 (1.4)	15.0 (1.4)
	Ethnicity, n(%)	Malay	416 (66.8)	189 (66.5)	227 (67.0)
		Chinese	78 (12.5)	43 (15.1)	35 (10.3)
		Indian	129 (20.7)	52 (18.3)	77 (22.7)
	Sex	Male	318 (51.0)	131 (46.1)	174 (51.3
		Female	305 (49.0)	153 (53.9)	165 (48.7)
	Household income (MYR)^a^	Median (IQR)	1500.00 (1000.0)	1500.0 (2000.0)	1500.0 (1000.0)
Food servings/day	Cereals and cereal products	Median (IQR)	5.8 (4.7)	5.6 (4.4)	5.6 (4.8)
	Fruits	Median (IQR)	3.0 (3.1)	1.9 (2.8)	1.9 (3.3)
	Vegetables	Median (IQR)	2.1 (2.7)	1.4 (2.1)	1.3 (2.5)
	Meat, poultry and eggs	Median (IQR)	1.7 (2.6)	1.4 (2.5)	1.9(2.9)
	Fish and seafood	Median (IQR)	1.0 (1.7)	1.0 (1.7)	1.0 (1.7)
	Legumes, seeds and nuts	Median (IQR)	0.7 (1.3)	0.7 (1.4)	0.7 (1.3)
	Milk and dairy products	Median (IQR)	1.0 (1.9)	1.0 (1.9)	0.9 (1.9)
	Processed foods and beverages	Median (IQR)	4.3 (5.2)	4.8 (5.0)	4.0 (5.2)
Blood pressure	Systolic blood pressure (mm/Hg)	Mean (SD)	110.2 (12.0)	106.2 (10.8)	113.5 (11.9)
	Diastolic blood pressure (mm/Hg)	Mean (SD)	66.7 (9.3)	65.4 (9.3)	67.7 (9.3)
Anthropometry	Height (cm)	Mean (SD)	149.2 (13.8)	138.6 (11.4)	158.0 (8.3)
	Weight (kg)	Mean (SD)	48.8 (18.2)	39.1 (14.5)	56.9 (16.9)
	BMI z score	Mean (SD)	0.6 (1.6)	0.8 (1.6)	0.5 (1.5)
	BMI category, n(%)	Underweight	24 (3.9)	8 (2.8)	16 (4.7)
		Healthy weight	348 (55.9)	150 (52.8)	198 (58.4)
		Overweight	106 (17.0)	45 (15.8)	61 (18.0)
		Obese	145 (23.3)	81 (28.5)	64 (18.9)
	Waist circumference (cm)	Mean (SD)	71.4 (15.6)	67.0 (14.8)	75.18 (15.3)
	Abdominal obesity, n (%)		57 (35.2)	28 (38.9)	29 (32.2)
Blood biomarkers^b^	Total cholesterol (mmol/L)	Mean (SD)	4.7 (0.7)	4.8 (0.7)	4.6 (0.8)
	Triglyceride (mmol/L)	Mean (SD)	1.0 (0.5)	1.0 (0.5)	1.0 (0.5)
	HDL cholesterol(mmol/L)	Mean (SD)	1.4 (0.2)	1.5 (0.3)	1.4 (0.3)
	LDL cholesterol (mmol/L)	Mean (SD)	2.8 (0.6)	2.8 (0.6)	2.8 (0.7)
	TC:HDL ratio	Mean (SD)	3.4 (0.8)	3.3 (0.7)	3.5 (0.9)
	Fasting plasma glucose (mmol/L)	Mean (SD)	4.7 (0.5)	4.7 (0.5)	4.7 (0.6)

^a^N = 614; ^b^N = 162.

Participants predominantly consumed cereals and cereal products, processed foods and beverages, and fruits and vegetables. However, a significant portion was overweight or obese, with 17 and 23.3% being overweight and obese, respectively. Abdominal obesity was found in 35.2% of the study participants. Overall, mean BPs, lipid profile, and FBG were normal.

### Comparison of dietary intake and adherence according to demography

In our analysis of median daily food group servings by demographics (Table 2), children had notably higher median consumption of processed foods and beverages than adolescents (4.8 servings vs. 4.0 servings, *p* = 0.044). Males consumed more cereals (6.5 servings vs. 4.8 servings, *p* < 0.001) but fewer vegetables (1.1 servings vs. 1.5 servings, *p* = 0.015) than females. Among ethnicities, Indians had the lowest intake of cereals, processed foods, and beverages, unlike Malays and Chinese. Chinese participants had the highest intake of vegetables, milk, and dairy, while Malays consumed more fish and seafood.

**Table 2:** Median daily servings of food intake compared by demographic characteristics.

Demography		n	Cereals & cereal products		Fruits		Vegetables		Meat, poultry & eggs	
			Median (IQR)	*P* value	Median (IQR)	*P* value	Median (IQR)	*P* value	Median (IQR)	*P* value
Age group	Children	284	5.6 (4.4)	0.503	1.9 (2.8)	0.562	1.4 (3.0)	0.374	1.4 (2.5)	0.090
	Adolescents	339	6.0 (4.8)		1.9 (3.3)		1.3 (2.5)		1.9 (2.9)	
Sex	Female	305	4.8 (4.2)	< 0.001**	2.1 (3.3)	0.392	1.5 (3.0)	0.015*	1.4 (2.4)	0.124
	Male	318	6.5 (4.6)		1.7 (3.0)		1.1 (2.6)		1.9 (3.0)	
Ethnicity^a^	Malay	416	6.2 (4.6)	< 0.001**	1.9 (3.4)	0.139	1.1 (2.7)	0.004*	1.9 (3.2)	< 0.001**
	Chinese	78	6.3 (4.2)		2.0 (2.7)		2.5 (3.0)		1.0 (1.9)	
	Indian	129	3.7 (3.7)		1.6 (2.2)		1.2 (2.5)		2.0 (2.1)	

Demography		n	Fish and seafood		Legumes, seeds & nuts		Milk & dairy products		Processed foods & beverages	
			Median (IQR)	*P* value	Median (IQR)	*P* value	Median (IQR)	*P* value	Median (IQR)	*P* value
Age group	Children	284	1.0 (1.7)	0.530	0.7 (1.4)	0.749	1.0 (1.9)	0.056	4.8 (5.0)	0.044*
	Adolescents	339	1.0 (1.7)		0.7 (1.3)		0.9 (1.9)		4.0 (5.2)	
Sex	Female	305	1.0 (1.8)	0.836	0.7 (1.1)	0.288	1.0 (1.9)	0.716	4.1 (5.0)	0.141
	Male	318	1.0 (1.5)		0.7 (1.4)		1.0 (1.9)		4.7 (5.5)	
Ethnicity^a^	Malay	416	1.2 (1.7)	< 0.001**	0.7 (1.4)	0.191	1.0 (1.8)	0.004*	4.8 (5.2)	< 0.001**
	Chinese	78	0.6 (1.3)		0.7 (1.6)		1.3 (3.0)		5.1 (6.2)	
	Indian	129	0.5 (1.1)		0.6 (1.0)		0.9 (1.6)		2.8 (3.5)	

^a^Post hoc analysis: Indian respondents consumed significantly lesser cereals and cereal products, and processed foods and beverages, than Malays and Chinese respondents (*p* < 0.001). Chinese respondents consumed significantly more vegetables (*p* < 0.05), and milk and dairy products (*p* < 0.05), but lesser meat, poultry and eggs (*p* = 0.001) than Malay and Indian respondents. Malay respondents consumed significantly more fish and seafood compared to Malays and Indian respondents (*p* < 0.05).

*significant at *p* < 0.05, **significant at *p* < 0.001.

**Figure 2:**
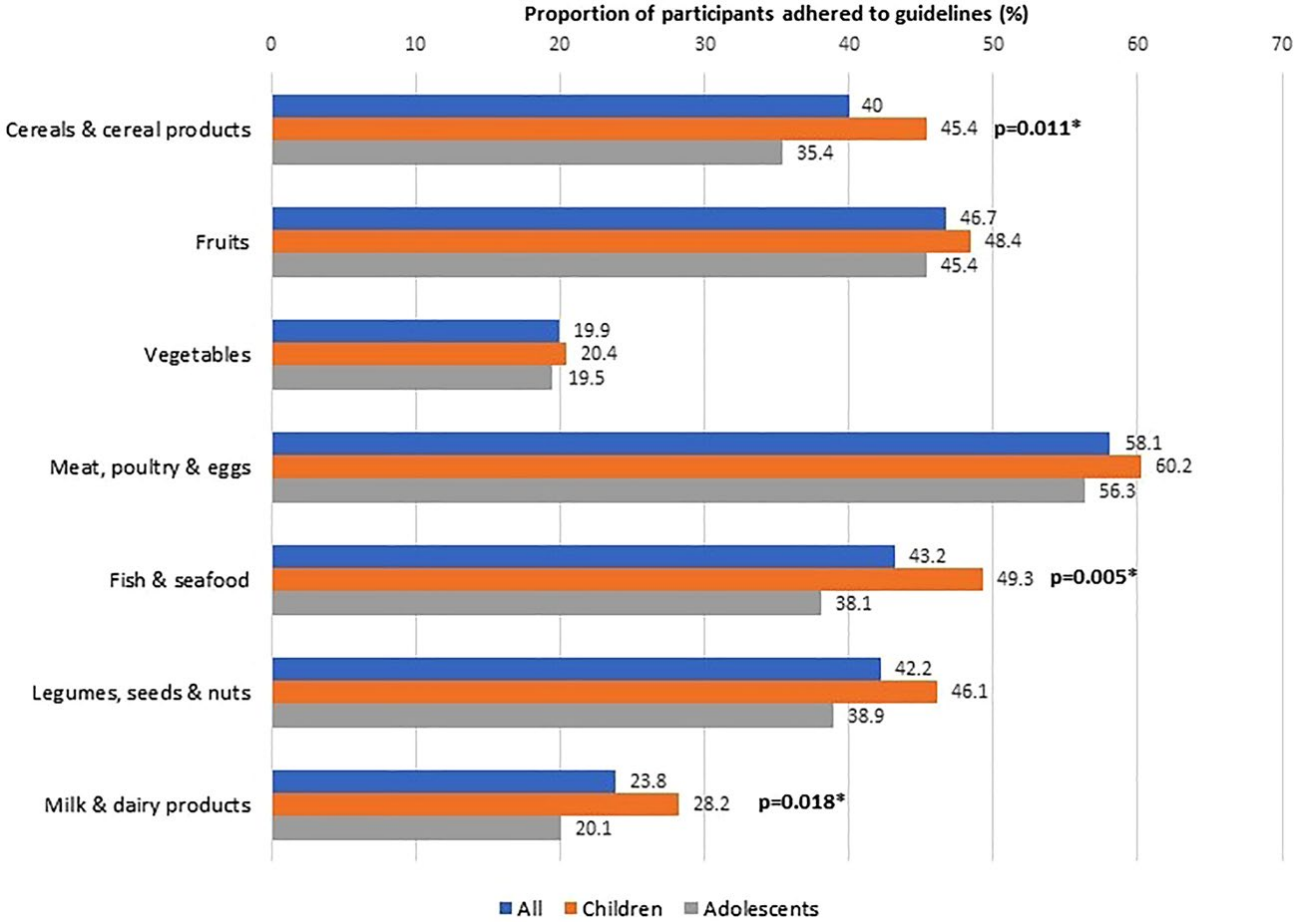
Adherence to dietary guidelines according to age groups (N = 623).

**Figure 3:**
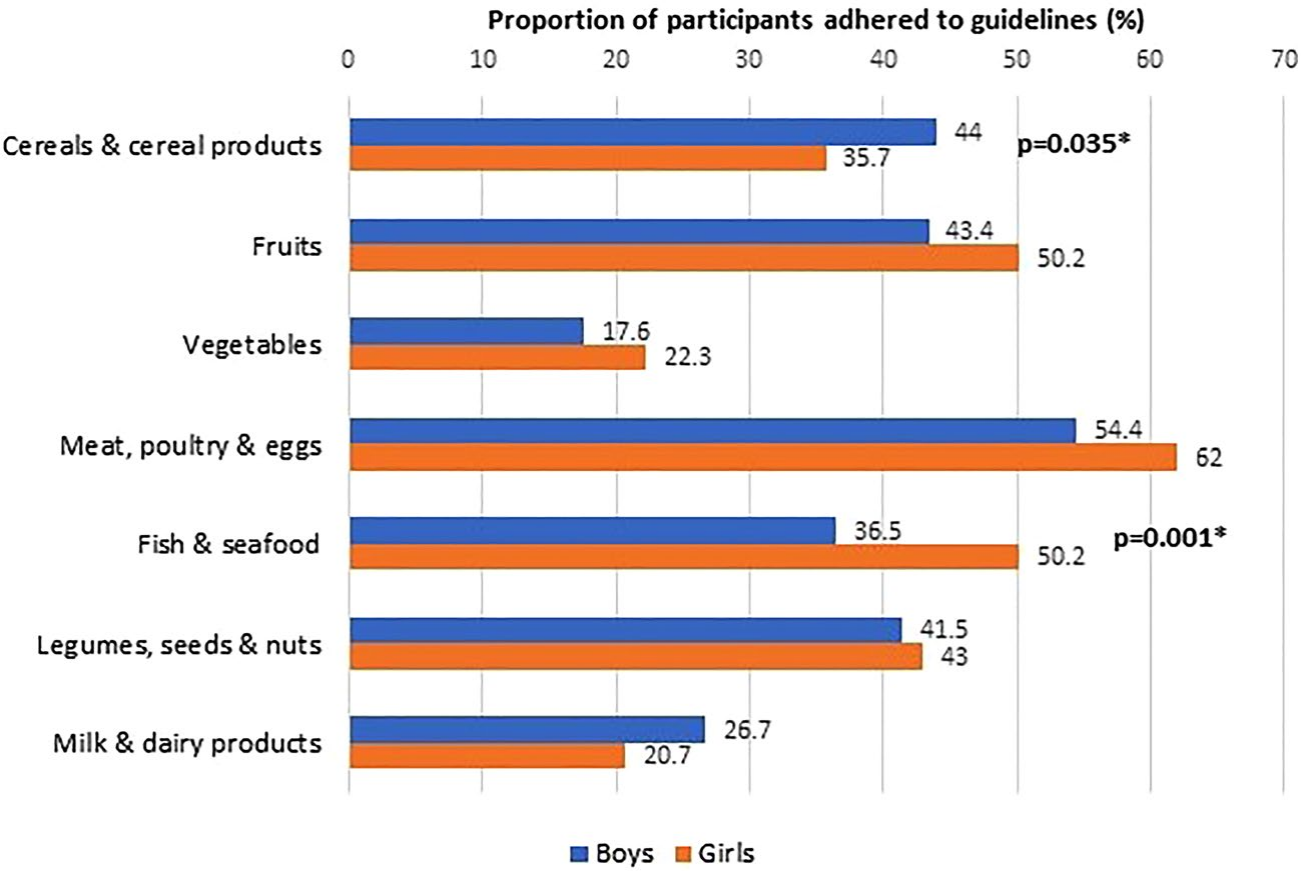
Adherence to dietary guidelines according to sexes (N = 623).

**Figure 4:**
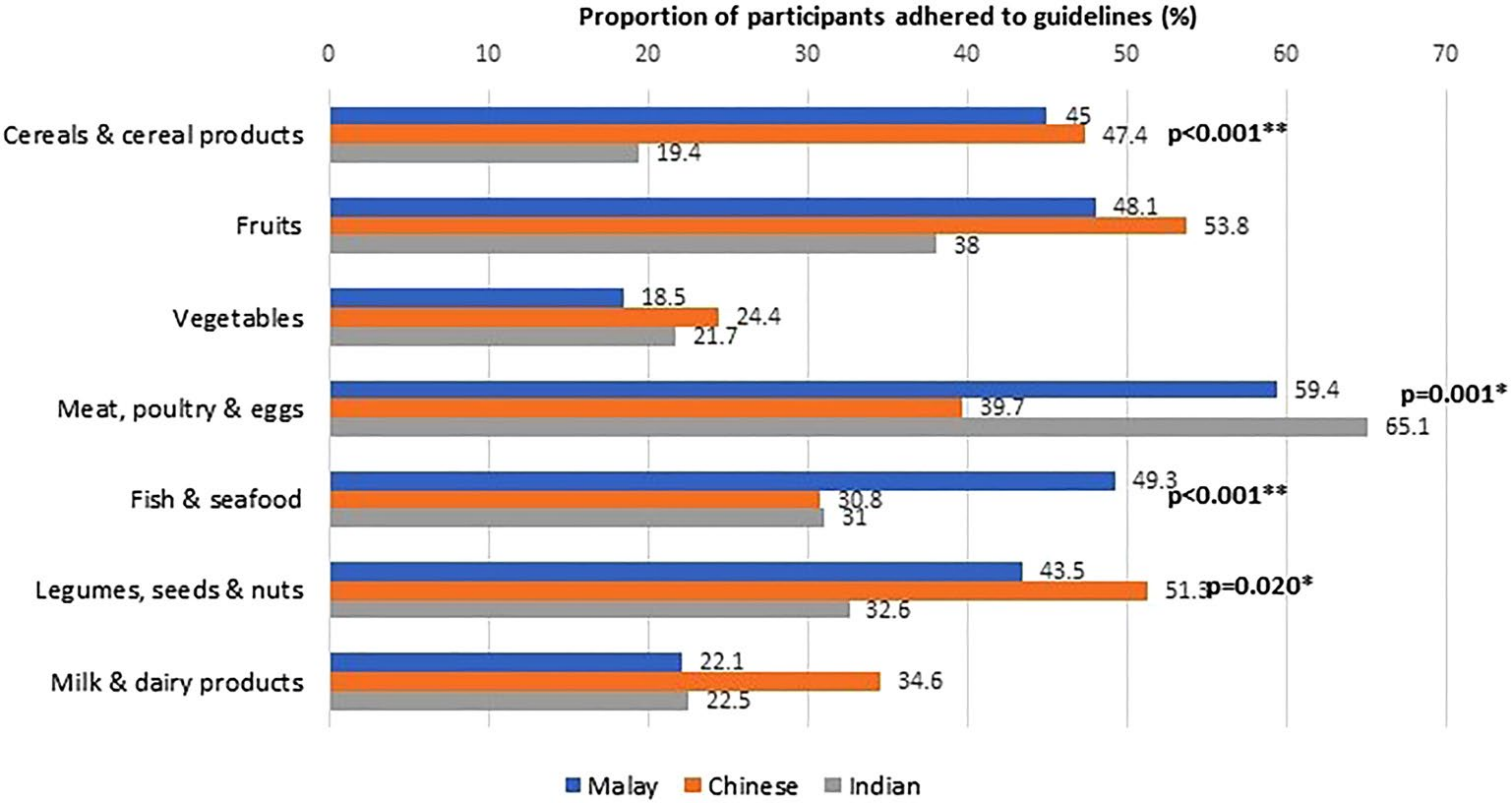
Adherence to dietary guidelines according to ethnicity (N = 623).

The adherence to age- and sex-specific dietary recommendations was generally low. Overall, the proportion of participants that adhered to recommended servings was below 50%, except for meat, poultry, and eggs (58.1%). Variations were noted in compliance with dietary guidelines, particularly for cereals and cereal products, fish and seafood, and milk and dairy products, with differences observed based on age, sex, and ethnicity (Figs. 2, 3 and 4).

### Comparison of dietary intake according to metabolic risk factors

Subsequently, we compared the median daily servings of food intake according to metabolic risk factors (Table 3) and explored the differences according to demographic sub-groups (Supplementary Tables S2, S3 and S4). Obese participants reportedly consumed more servings of fruits than those with normal weight (2.2 servings/day vs. 1.7 servings/day, *p* = 0.034). Fruit consumption was also higher among abdominally obese children, males, and Chinese participants (Supplementary Tables S2, S3 and S4).

**Table 3:** Median daily servings of food intake compared by metabolic risk factors.

Metabolic risk factors		n	Cereals & cereal products		Fruits		Vegetables		Meat, poultry & eggs	
			Median (IQR)	*P* value	Median (IQR)	*P* value	Median (IQR)	*P* value	Median (IQR)	*P* value
Obesity	Yes	348	5.8 (4.4)	0.726	2.2 (3.7)	0.034*	1.3 (2.6)	0.561	1.7 (2.7)	0.846
	No	251	5.7 (4.9)		1.7 (2.7)		1.3 (2.8)		1.9 (2.7)	
Abdominal obesity	Yes	151	6.2 (4.8)	0.717	2.1 (4.6)	0.122	1.6 (3.0)	0.118	1.6 (2.9)	0.835
	No	472	5.7 (4.6)		1.8 (2.7)		1.2 (2.7)		1.9 (2.7)	
Elevated blood pressure	Yes	41	6.4 (6.1)	0.270	1.4 (4.0)	0.418	1.0 (1.9)	0.353	1.7 (2.6)	0.799
	No	582	5.8 (4.6)		1.9 (3.0)		1.3 (2.7)		1.7 (2.8)	
Elevated triglyceride	Yes	13	6.8 (5.6)	0.598	0.8 (3.6)	0.198	1.0 (2.5)	0.794	1.4 (3.7)	0.948
	No	148	5.6 (4.7)		1.6 (2.9)		1.2 (2.7)		2.0 (2.9)	
Low HDL cholesterol	Yes	13	5.9 (5.6)	0.897	1.9 (4.7)	0.946	1.0 (2.6)	0.873	4.0 (3.8)	0.011*
	No	149	5.6 (4.6)		1.5 (2.7)		1.3 (2.6)		1.7 (2.9)	
Elevated fasting blood glucose	Yes	5	6.8 (4.9)	0.513	0.8 (2.3)	0.280	0.3 (3.8)	0.498	5.0 (4.7)	0.111
	No	157	5.6 (4.7)		1.6 (3.0)		1.3 (2.6)		2.0 (2.9)	
Metabolic syndrome	Yes	7	5.4 (3.9)	0.436	0.8 (5.4)	0.249	2.3 (3.6)	0.277	4.0 (5.3)	0.247
	No	155	5.6 (4.7)		1.6 (2.9)		1.1 (2.6)		2.0 (2.9)	

Metabolic risk factors		n	Fish and seafood		Legumes, seeds & nuts		Milk & dairy products		Processed foods & beverages	
			Median (IQR)	*P* value	Median (IQR)	*P* value	Median (IQR)	*P* value	Median (IQR)	*P* value
Obesity	Yes	348	1.0 (1.6)	0.746	0.7 (1.4)	0.378	0.9 (2.3)	0.537	4.1 (4.9)	0.390
	No	251	1.0 (1.7)		0.7 (1.3)		1.00 (1.7)		4.4 (5.3)	
Abdominal obesity	Yes	151	1.2 (1.7)	0.453	0.7 (1.3)	0.686	0.8 (2.2)	0.165	4.0 (4.6)	0.268
	No	472	0.9 (1.7)		0.7 (1.4)		1.0 (1.7)		4.5 (5.3)	
Elevated blood pressure	Yes	41	0.9 (1.0)	0.124	0.6 (1.4)	0.906	0.5 (0.9)	0.008*	3.3 (3.5)	0.003*
	No	582	1.0 (1.7)		0.7 (1.3)		1.0 (2.0)		4.4 (5.3)	
Elevated triglyceride	Yes	13	0.5 (1.4)	0.146	0.5 (1.1)	0.414	0.8 (1.8)	0.904	3.0 (5.8)	0.250
	No	148	1.0 (1.7)		0.8 (1.3)		1.1 (2.2)		4.7 (4.9)	
Low HDL cholesterol	Yes	13	1.1 (1.8)	0.271	1.7 (1.9)	0.344	1.4 (3.7)	0.159	6.2 (5.6)	0.882
	No	149	1.0 (1.6)		0.7 (1.1)		1.0 (2.2)		4.5 (4.7)	
Elevated fasting blood glucose	Yes	5	2.1 (5.3)	0.735	0.6 (0.6)	0.203	1.8 (5.4)	0.628	6.1 (10.4)	0.391
	No	157	1.0 (1.6)		0.8 (1.3)		1.1 (2.2)		4.4 (4.8)	
Metabolic syndrome	Yes	7	0.4 (2.1)	0.099	0.5 (1.8)	0.630	0.8 (0.8)	0.561	1.9 (3.3)	0.094
	No	155	1.0 (1.6)		0.8 (1.2)		1.1 (2.2)		4.7 (4.8)	

*significant at *p* < 0.05.

N = 623 except for obesity (underweight subjects were excluded) and biomarkers (a subset of respondents underwent laboratory investigations).

Overall, participants with elevated BP had a lower intake of milk and dairy products (0.5 servings/day vs. 1.0 servings/day, *p* = 0.008) and a lower intake of processed foods and beverages (3.3 servings/day vs. 4.4 servings/day, *p* = 0.003), compared to participants with normal BP levels (Table 3). Sub-group analysis showed similar differences persisted among adolescents, males, and Indian participants (Supplementary Tables S2, S3 and S4).

Female participants with elevated TG reported a lower intake of fruits (0.8 servings/day vs. 1.2 servings/day, *p* = 0.011) but a higher intake of fish and seafood (2.3 servings/day vs. 0.9 servings/day, *p* = 0.042) (Supplementary Table S3), while those with low HDL-C levels consumed more meat, poultry, and eggs (1.7 servings/day vs. 4.0 servings/day, *p* = 0.011) (Table 3). The finding was reaffirmed among adolescents, males, and Indian participants (Supplementary Tables S2, S3 and S4).

Children with elevated FBG levels tended to consume fewer vegetables (0.3 servings/day vs. 1.5 servings/day, *p* = 0.021), while adolescents consumed more meat, poultry, and eggs (6.1 servings/day vs. 1.7 servings/day, *p* = 0.040) compared to their counterparts with normal FBG levels (Supplementary Table S2). The findings, however, need to be treated with caution due to the small number of participants with elevated FBG.

None of the food groups were associated with overall MetS (Table 3). However, the sub-group analyses showed lower consumption of fruits among females with MetS (0.2 servings/day vs. 1.3 servings, *p* = 0.008) (Supplementary Table S3) and higher consumption of meat, poultry, and eggs among Indian participants with MetS (7.4 servings/day vs. 2.0 servings/day, *p* = 0.011) (Supplementary Table S4) than their counterparts without MetS.

Anthropometric and biomarker associations with dietary recommendations (Supplementary Table S5) revealed that inadequate consumption of cereals and cereal products was associated with higher median WC (70.0 cm vs. 67.5 cm, *p* = 0.030), while adherence to fish and seafood intake recommendations was associated with lower median SBP (107 mmHg vs. 110 mmHg, *p* = 0.001). Conversely, adherence to milk and dairy products was associated with a higher BMI z-score (0.9 vs. 0.4, *p* = 0.028).

## Discussion

We analysed the dietary data and metabolic indicators of 623 participants enrolled in SEACO-CH20, comprising 285 children (aged 7–12) and 389 adolescents (aged 13–18). Most respondents were Malay, with almost equal proportions of males and females. Cereals and cereal products, processed foods and beverages, and fruits and vegetables were the most consumed food groups. The prevalence of overweight/obesity, and abdominal obesity in this population was worrying, with at least one in three affected. Adherence to dietary guidelines was generally low, except for meat, poultry, and eggs, though we noted differences in food consumption based on age, sex, and ethnicity. Some paradoxical associations between food groups and health markers, such as fruit consumption and obesity were also found.

In our study, children demonstrated higher adherence to dietary guidelines for cereals and cereal products, fish and seafood, and milk and dairy products compared to adolescents. The SEANUTS study^32^ further revealed that while younger children (7–9 years) were more likely to meet recommendations for cereals/grains, older children consumed a greater number of servings of cereals/grains, vegetables, meat/poultry, fish, and legumes. Conversely, there was a notable trend among SEACO-CH20 children towards increased consumption of processed foods and beverages, aligning with previous research highlighting their preference for such foods^33,34^. Evidence from recent systematic reviews underscores the association between the intake of ultra-processed foods and the risk of obesity and adiposity in children^34^. Additionally, sugars and sweet products emerged as the most favored processed foods among children^35^. These findings emphasize the necessity for early dietary education interventions, particularly given children’s susceptibility to less healthy food choices.

The current study also explored the ethnic-differences in food intake. Children and adolescents of Indian ethnicity reported the lowest intake of cereals and cereal products as well as processed foods and beverages. Indians also reported relatively higher adherence to meat, poultry and eggs recommendations, a finding that contradicted previously reported SEANUTS report^32^. Chinese respondents in SEACO-CH20 reported relatively higher consumption of vegetables, milk, and dairy products. They also more frequently adhered to recommendations for cereals and cereal products, legumes, nuts and seeds. On the other hand, Malays preferred and had higher adherence to fish and seafood. Several studies have explored ethnic variations in adult food intake, though the evidence among children and adolescents is limited. For example, Garba et al.^36^ reported ethnicity as a predictor of adolescent diet, especially dietary patterns associated with fruits, vegetables, fats and sugar. Findings from the Malaysian Adolescent Nutrition Survey 2017 also support an association between ethnicity and unhealthy dietary patterns characterised by foods with high sugar content, oil or fat, salt, and processed foods^37^.

We also found a disparity in food intake between the sexes. Male SEACO-CH20 respondents consumed more cereals and cereal products and showed better adherence to the recommended servings. However, they consumed fewer servings of fruits and vegetables than female participants. Female respondents showed higher compliance with guidelines for fish and seafood intake. Several studies have explored sex-related differences in dietary intake. For example, the VYRONAS study^38^ reported a preference for cereals and cereal products, especially for breakfast by boys aged 12–17 compared to their female counterparts. This likely reflects the higher need for foods that contribute more energy during rapid growth in boys. In addition, lower fruit and vegetable intake among the boys also corresponds to some previous findings. Boys have been reported to consume fewer fruits and vegetables because they like them less and have a greater liking for energy-dense foods^39,40^. However, this is not consistent, as a number of studies did not report a sex difference in vegetable consumption, warranting further exploration^41^.

We uncovered a surprising and intriguing result where overweight/obese participants consumed more servings of fruits than those with normal weight. Our sub-group analysis revealed higher fruit consumption among abdominally obese children, males, and Chinese participants. This challenges traditional assumptions about the protective effect of fruits and warrants further investigations. Sharma and colleagues^42^ suggested the effect of certain types of fruits and the increase in simple sugars as potential factors for the contradictory effects of fruits on obesity.

Participants with elevated BP consumed fewer servings of milk and dairy products. Although the median consumption (1 serving/day) is still below the recommended servings, the protective effect of milk and dairy products corresponds to previous studies. The QUALITY cohort, for example, indicated that high consumption of dairy products has antihypertensive effects on children^43^. However, we discovered another surprising finding with a lower intake of processed foods and beverages with elevated BP. The level of processing of the foods could have influenced the BP. However, the current data did not allow for further subgroup analysis according to the level of processing. Similar to the contradictory effect of fruit-obesity, this paradoxical relationship also should be investigated further.

The SEACO-CH20 finding showing female children and adolescents with elevated TG reported higher fish and seafood consumption, but lower fruit intake is noteworthy. These sex-specific relationships may have implications for managing TG levels and subsequent cardiovascular health. As the evidence has been contradictory^44^, further investigation should explore the type of fish and seafood that influence blood TG levels. Evidence associating fruit intake and TG is also scarce and inconsistent, meriting further exploration^45^.

Low HDL-C levels were associated with a higher intake of animal protein sources, and the effect was specifically seen in adolescents, males and Indian participants of this current study. The negative association between animal protein sources or dietary patterns high in animal sources and low HDL-C in the paediatric population has been previously documented in large-scale surveys^46,47^. Higher intake of meat, poultry and eggs was also found among adolescents with elevated levels of FBG in our study.

Although there is an absence of significant associations between food groups and MetS, sub-group analysis showed that females with MetS consumed fewer servings of fruits. In addition, Indian participants with MetS reported higher consumption of meat, poultry and eggs than their counterparts without MetS. Western dietary patterns, which are generally high in animal protein but low in fruits and vegetables, have been associated with the risk for MetS in the past^48^. A meta-analysis suggests a higher consumption of fruits was associated with lower odds for MetS among Asians, although the findings were not specific to adolescents or females^49^. Data from a large nationwide survey in China showed lower fruit intake was associated with some components of metabolic diseases which were more evident among female adolescents. However, the researchers did not associate the fruit intake with MetS itself^50^.

We discovered additional insights into the relationship between dietary intake and metabolic indicators by evaluating adherence to dietary guidelines. Children and adolescents who adhered to the recommended servings of milk and dairy products had higher BMI z-scores. However, the z-score was within the normal range. Hence there is no metabolic risk indicated. Adherence to recommended servings of fish and seafood suggested lower systolic BP, while adherence to cereals and cereal product recommendations suggested lower waist circumference. While optimal intake of fish, seafood, cereals and cereal products may have a positive effect on metabolic health, the impact of the type of foods, including fatty fish and whole grains needs to be investigated further.

The overall dietary intake of the SEACO-CH20 children and adolescents was found to be suboptimal, with less than 50% meeting dietary recommendations across all food groups except for meat, poultry, and eggs (Fig. 2). This trend persists when examining the data by sex (Fig. 3) and ethnicity (Fig. 4). Although this study did not analyze food consumption patterns, there are indications that the participants, particularly the adolescents, may be inclined towards unhealthy dietary habits. Recent local studies have highlighted poor dietary practices and patterns among Malaysian children and adolescents, which have been linked to a poor quality of life^51–53^. Moreover, unhealthy dietary habits established at a young age are associated with an increased risk of cardiometabolic diseases and poor quality of life^52–56^. These age-, sex- and ethnic-specific variations in our SEACO-CH20 study highlight the importance of tailoring nutritional interventions to address the unique dietary preferences and cultural contexts of different ethnic communities. Interventions to promote healthy eating habits among children and adolescents, such as school-based nutrition education programs, parental involvement, and policy changes to improve access to nutritious foods in schools’ canteens, are crucial for long-term health outcomes^57^. Cultural beliefs and family dynamics also play a significant role in shaping dietary choices among young individuals, highlighting the need for culturally tailored interventions targeting this age group^58,59^.

The current study demonstrates several strengths. We recruited a sizable (N = 623) and diverse paediatric participants comprising an equal number of females and males. This provides a comprehensive representation of the study population in SEACO. In addition, the large sample enhances the generalisability of the findings to the children and adolescent population in Malaysia. Although the number of participants available for the biomarker subset was relatively smaller, we were able to perform sub-group analyses exploring the possible association between dietary intake and metabolic outcomes.

However, this study also exhibits several limitations. The study’s cross-sectional design did not allow for establishing a causal relationship between food intake and metabolic indicators. Future studies should focus on a longitudinal design to provide a clearer understanding of the temporal relationship between these factors. The number of samples available for blood biomarker analysis was significantly lower than the total number of study participants, which could also undermine the strength of the relationship. Owing to the impact of the COVID-19 pandemic, the Malaysian government’s enforcement of movement control measures impeded the recruitment of participants with blood samples to achieve the initially proposed sample size. Consequently, the attained response rate amounted to 23%. This is also a reason why we are not able to control potential confounders. However, we conducted sub-group analyses to explore the effect of these confounders on the association between food intake and metabolic risk factors. We did not correct for multiple testing as the sub-group comparisons were supplementary to the primary hypothesis of assessing the link between demography, metabolic risk factors, and dietary intake (food groups). However, future studies exploring the associations specific to certain demography should consider multiple comparisons in study designs or adjust for multiple testing at the data analysis stage. One limitation of this study is the potential for implausible dietary data reporting among children and adolescents. Dietary data used in this analysis were self-reported and collected using a brief semi-FFQ instrument, which introduces a potential bias that could affect the accuracy of the information. Despite efforts to minimise the bias through standardised methodologies, the possibility of inaccuracies remains. Incorporating dietary biomarkers could be an option for researchers aiming for a more objective assessment of dietary intake in the future. In addition, This study faces another significant limitation stemming from the timing of data collection, which coincided with the COVID-19 pandemic. The pandemic likely influenced participants’ dietary habits and their accuracy in reporting them.

## Conclusion

In this paper, we described the dietary intake of a multi-ethnic paediatric population during the recovery period of the COVID-19 pandemic. The study also examined the associations between food intake and metabolic risk factors such as obesity, abdominal obesity, elevated BP, dyslipidaemia and increased FBG. The study resulted in both supportive and contradictory findings in this subject matter. Despite a number of limitations, SEACO-CH20 study’s strengths lie in its large sample size and sub-group analyses. This study contributes valuable knowledge on the complex relationship between diet and metabolic health, especially in an under-studied paediatric population in Malaysia.

### Supplementary Information

Supplementary Information.

## Data Availability

All data generated or analysed during this study are included in this published article [and its supplementary information files]. Data are available from SEACO by completion of a data application form to: https://www.monash.edu.my/seaco/research-and-training/how-to-collaborate-with-seaco. For the purpose of open access, the author(s) has applied a Creative Commons Attribution (CC BY) licence to any Author Accepted Manuscript version arising from this submission.
