# Supplementary material for: Dietary intake, obesity, and metabolic risk factors among children and adolescents in the SEACO-CH20 cross-sectional study
Source: Sci Rep. 2024 May 17;14:11265. doi: 10.1038/s41598-024-61090-7 (PMC11101611; doi:10.1038/s41598-024-61090-7)
Supplement: Supplementary file 1 — Supplementary Information. [file 41598_2024_61090_MOESM1_ESM.pdf]

Supplementary Table S1: Food groups and corresponding food items

**Food group**	**Food items**
Cereals and cereal products	Rice (all rice varieties), nasi lemak, rice porridge, rice noodles / laksa, yellow / wheat noodles, bread (slices, all varieties), sweet buns (all varieties), *thosai*, *roti canai, capati, putu mayang, idli,* potatoes (all varieties)
Meat, poultry and eggs	Meat and poultry (all types), eggs (all types)
Fish and seafood	Fish (all types), squid, prawns, cockles (all varieties), anchovies
Milk and dairy products	Milk (all types), milk powder (all types), yogurt, *dadih,* cheese
Legumes, nuts and seeds	Tempe, tauhu, soy milk, all nuts and seeds, dhal (all varieties), canned beans
Fruits	Fresh whole fruits, fresh sliced fruits, durian, grapes
Vegetables	Green vegetables (cooked), salad*/ulam* (raw), fruit vegetables (cooked), legume vegetables (cooked)
Processed foods and beverages	Local *kuih*, cakes, doughnuts, pastries, chocolate, sweets, ice cream, condensed milk, malt beverages, other beverages, burger, sausages, french fries, pizza, butter/margarine, jam, kaya, peanut butter/chocolate spread

Supplementary Table S2: Median daily servings of food intake compared by metabolic risk factors according to age groups.

Metabolic risk factors	Age group		n	Cereals & cereal products		Fruits		Vegetables		Meat, poultry & eggs		Fish and seafood		Legumes, seeds & nuts		Milk & dairy products		Processed foods & beverages	
				Median (IQR)	P value	Median (IQR)	P value	Median (IQR)	P value	Median (IQR)	P value	Median (IQR)	P value	Median (IQR)	P value	Median (IQR)	P value	Median (IQR)	P value
Obesity	Children	Yes	126	5.7 (4.8)	0.405	2.3 (3.4)	0.128	1.3 (2.8)	0.291	1.4(2.5)	0.630	1.0 (1.6)	0.736	0.7 (1.6)	0.378	1.1(1.7)	0.854	4.4 (4.9)	0.659
		No	150	5.6 (4.6)		1.5 (2.6)		1.5 (3.1)		1.6 (2.7)		0.9 (1.7)		0.7 (1.2)		1.0 (2.3)		4.8 (5.2)	
	Adolescents	Yes	125	5.8 (4.1)	0.749	2.1 (4.0)	0.155	1.3 (2.5)	0.861	2.0 (2.9)	0.961	0.9 (1.6)	0.455	0.7 (1.3)	0.728	0.6 (2.2)	0.410	3.8 (4.4)	0.333
		No	198	5.8 (5.1)		1.8 (2.7)		5.7 (2.6)		1.9 (2.7)		1.0 (1.5)		0.6 (1.3)		0.9 (1.8)		3.8 (5.4)	
Abdominal obesity	Children	Yes	62	6.5 (5.3)	0.132	2.4 (4.4)	**0.017***	1.8 (3.1)	0.488	1.4 (2.4)	0.943	1.1 (1.6)	0.586	0.8 (1.5)	0.288	1.0 (2.5)	0.835	5.0 (5.0)	0.580
		No	222	5.5 (4.1)		1.6 (2.7)		1.4 (2.9)		4.7 (2.6)		0.9 (1.7)		0.7 (1.3)		1.0 (1.8)		4.7 (5.0)	
	Adolescents	Yes	89	6.1 (4.0)	0.393	1.6 (4.2)	0.998	1.6 (2.8)	0.118	1.9 (3.0)	0.795	1.2 (1.8)	0.611	0.6 (1.0)	0.147	0.5 (2.2)	0.124	3.6 (3.8)	0.084
		No	250	5.9 (4.9)		1.9 (3.0)		1.1 (2.5)		1.9 (2.8)		0.9 (1.6)		0.7 (1.4)		1.0 (1.8)		4.3 (5.5)	
Elevated blood pressure	Children	Yes	9	6.5 (6.3)	0.224	2.1 (8.3)	0.979	1.0 (1.8)	0.313	1.2(3.4)	0.789	1.0 (1.1)	0.723	1.1 (2.3)	0.290	0.6 (1.2)	0.468	3.3 (4.7)	0.079
		No	275	5.6 (4.4)		1.9 (2.8)		1.4 (3.1)		1.4 (2.5)		1.0 (1.7)		0.7 (1.3)		1.0 (2.0)		4.9 (4.9)	
	Adolescents	Yes	32	6.4 (6.3)	0.621	1.4 (3.9)	0.391	1.1(2.1)	0.699	1.7 (2.4)	0.844	0.7 (1.1)	**0.039***	0.6 (1.2)	0.505	0.5 (0.9)	**0.020***	3.2 (2.4)	**0.041***
		No	307	5.9 (4.7)		1.9 (3.2)		1.3 (2.6)		1.9 (2.9)		1.0 (1.7)		0.7 (1.3)		0.9 (2.0)		4.1 (5.4)	
Elevated triglyceride	Children	Yes	5	4.6 (7.2)	0.667	0.7 (3.2)	0.088	0.6 (4.0)	0.932	1.0 (4.6)	0.575	0.4 (1.9)	0.088	1.1 (1.2)	0.715	0.8 (4.9)	0.780	5.0 (11.2)	0.731
		No	67	5.6 (4.3)		2.1 (2.6)		1.4 (2.7)		2.3 (3.1)		1.4 (2.5)		0.7 (1.3)		1.6 (2.4)		5.1 (5.1)	
	Adolescents	Yes	8	7.7 (5.3)	0.366	1.5 (4.4)	0.736	1.4 (1.6)	0.631	1.9 (3.2)	0.523	0.7 (1.5)	0.752	0.4 (0.7)	0.156	0.9 (0.6)	0.920	2.8 (4.9)	0.326
		No	81	5.8 (4.7)		1.2 (3.0)		1.1 (2.5)		1.7 (1.7)		0.9 (1.1)		0.8 (1.3)		0.9 (2.1)		3.9 (5.0)	
Low HDL cholesterol	Children	Yes	3	4.9	1.000	1.0	**0.024***	1.0	0.895	3.1	0.151	3.1	0.177	2.1	**0.024***	4.2	0.159	7.5	0.216
		No	69	5.6 (4.3)		1.8 (2.5)		1.4 (2.8)		2.1 (3.1)		1.4 (2.4)		0.6 (1.4)		1.5 (2.4)		5.0 (5.2)	
	Adolescents	Yes	10	6.3 (5.4)	0.908	1.0 (2.7)	0.426	1.5 (2.5)	0.959	4.1 (4.2)	**0.042***	1.1 (1.6)	0.320	0.5 (2.0)	0.743	1.2 (3.3)	0.243	2.8 (5.4)	0.832
		No	80	5.8(4.7)		1.3 (3.2)		1.1 (2.4)		1.4 (2.2)		0.9 (1.0)		0.8(1.1)		0.8 (1.8)		3.9 (5.1)	
Elevated fasting blood glucose	Children	Yes	3	8.6	0.135	1.3	0.750	0.3	**0.021***	1.6	0.770	2.1	0.650	0.6	0.537	1.8	1.000	6.1	0.592
		No	69	5.5 (4.3)		1.9 (2.7)		1.5 (2.7)		2.3 (3.1)		1.4 (2.5)		1.0 (1.3)		1.5 (2.4)		5.1 (5.2)	
	Adolescents	Yes	2	4.6	0.464	0.5	0.144	4.0	0.120	6.1	**0.040***	1.2	0.902	0.4	0.300	3.7	0.612	9.8	0.799
		No	88	5.9 (4.9)		1.3 (3.2)		1.1 (2.3)		1.7 (2.5)		0.9 (1.1)		0.8 (1.3)		0.9 (1.8)		3.9 (5.0)	
Metabolic syndrome[a]	Adolescents	Yes	7	5.4 (3.9)	0.438	0.8 (5.4)	0.346	2.3 (3.6)	0.214	4.0 (5.3)	0.252	0.4 (2.1)	0.178	0.5 (1.8)	0.527	0.8 (0.8)	0.845	1.9 (3.3)	0.206
		No	83	5.9 (5.0)		1.3 (2.8)		1.1 (2.3)		1.7 (2.3)		1.0 (1.1)		0.8 (1.3)		0.9 (1.9)		3.9 (4.9)	

*significant at $p<0.05$

N=284 (children) and N=339 (adolescents), except for obesity (underweight subjects were excluded) and biomarkers (a subset of respondents underwent laboratory investigations).

[a]No children were found to have metabolic syndrome.

IQR not provided for n<4

Supplementary Table S3: Median daily servings of food intake compared by metabolic risk factors according to sexes.

Metabolic risk factors	Sex		n	Cereals & cereal products		Fruits		Vegetables		Meat, poultry & eggs		Fish and seafood		Legumes, seeds & nuts		Milk & dairy products		Processed foods & beverages	
				Median (IQR)	P value	Median (IQR)	P value	Median (IQR)	P value	Median (IQR)	P value	Median (IQR)	P value	Median (IQR)	P value	Median (IQR)	P value	Median (IQR)	P value
Obesity	Female	Yes	111	5.2 (4.4)	0.247	2.5 (4.1)	0.030*	1.7 (2.8)	0.756	1.4 (2.6)	0.872	1.0 (1.7)	0.893	0.7 (1.5)	0.236	0.8 (2.8)	0.790	4.0(4.4)	0.978
		No	185	4.4 (4.2)		1.8 (2.7)		1.4 (3.0)		1.7 (2.4)		1.0 (1.8)		0.6 (1.0)		1.0 (1.7)		1.0 (5.2)	
	Male	Yes	140	6.3 (4.6)	0.176	1.9 (3.3)	0.328	1.2 (2.4)	0.820	2.0 (2.8)	0.810	1.0 (1.8)	0.712	0.7 (1.4)	0.915	0.9 (2.1)	0.473	4.3 (5.0)	0.176
		No	163	6.7 (4.3)		1.6 (2.7)		1.1 (2.7)		1.9 (3.3)		1.0 (1.6)		0.7 (1.5)		1.0 (1.9)		4.9 (6.2)	
Abdominal obesity	Female	Yes	86	5.2 (4.4)	0.434	1.7 (4.0)	0.753	1.9 (3.0)	0.494	1.4 (2.3)	0.720	1.0 (1.6)	0.884	0.7(1.1)	0.608	0.8 (2.4)	0.341	3.6 (3.8)	0.266
		No	219	4.6 (4.1)		2.1 (3.0)		1.4 (2.9)		1.6 (2.4)		1.0 (1.8)		0.7 (1.2)		1.0 (1.8)		4.3 (5.3)	
	Male	Yes	65	6.8 (4.7)	0.606	2.4 (4.7)	**0.008***	1.4 (3.0)	0.215	1.7 (3.0)	0.880	1.3 (1.9)	0.195	0.8 (1.5)	0.939	0.8 (2.1)	0.326	4.4 (5.4)	0.861
		No	253	6.5 (4.6)		1.5 (2.6)		1.1 (2.6)		2.0 (3.1)		0.9 (1.5)		0.7 (1.4)		1.0 (1.9)		4.7 (5.5)	
Elevated blood pressure	Female	Yes	15	4.4 (4.4)	0.901	2.3 (4.1)	0.999	1.0 (1.7)	0.302	1.6(3.4)	0.165	0.8 (1.6)	0.418	0.6 (1.6)	0.848	0.5 (0.4)	**0.010***	2.6 (3.5)	0.059
		No	290	4.8 (4.2)		2.1 (3.2)		1.6 (3.1)		0.9 (1.6)		1.0 (1.8)		0.7 (1.1)		1.0 (2.0)		4.1 (5.0)	
	Male	Yes	26	7.5 (5.3)	0.264	1.1 (4.4)	0.301	1.0 (2.1)	0.819	2.1 (2.5)	0.240	0.9 (1.1)	0.192	0.7 (1.4)	0.920	1.0 (1.0)	**0.163**	3.6 (3.3)	**0.016***
		No	292	6.5 (4.5)		1.8 (2.9)		1.2 (2.6)		1.9 (3.2)		1.0 (1.7)		0.7 (1.5)		1.0 (2.1)		4.8 (5.5)	
Elevated triglyceride	Female	Yes	3	8.6	0.757	0.8	**0.011***	0.3	0.750	5.0	0.193	2.3	**0.042***	0.2	0.841	4.0	0.884	8.6	0.444
		No	73	4.5 (4.2)		1.2 (2.6)		1.4 (3.0)		1.4 (2.3)		0.9 (1.4)		0.6 (1.1)		1.1 (2.1)		3.3 (4.7)	
	Male	Yes	2	4.5	0.456	1.0	0.466	1.3	0.471	3.9	0.095	1.1	0.735	0.7	0.401	1.0	0.812	3.0	0.421
		No	84	6.7 (4.6)		2.0 (3.6)		1.1 (2.4)		2.0 (3.1)		1.3 (1.8)		0.9 (1.4)		1.1 (2.4)		5.0 (5.0)	
Low HDL cholesterol	Female	Yes	6	4.7 (6.2)	0.946	2.5 (6.0)	0.610	1.4 (4.0)	0.679	5.0 (7.4)	0.157	2.3 (4.2)	0.133	1.1 (1.9)	0.397	3.1 (4.5)	0.059	4.6(10.1)	0.513
		No	70	4.7 (4.4)		1.2 (2.4)		1.4 (3.0)		1.4 (2.2)		0.8 (1.3)		0.5 (1.1)		0.9 (2.2)		3.7 (4.8)	
	Male	Yes	7	6.8 (4.6)	0.906	1.9 (4.8)	0.676	1.0 (1.8)	0.850	4.0 (1.9)	**0.042***	1.0 (1.4)	0.962	1.7 (2.1)	0.564	1.1 (3.0)	0.856	6.2 (5.6)	0.636
		No	79	6.7 (4.8)		1.9 (3.2)		1.1 (2.4)		2.0 (3.1)		1.3 (1.8)		0.9 (1.3)		1.0 (2.2)		4.9 (5.1)	
Elevated fasting blood glucose	Female	Yes	3	8.6	0.061	0.8	0.613	0.3	0.559	5.0	0.150	2.3	0.354	0.2	0.175	4.0	0.382	8.6	**0.035***
		No	73	4.5 (4.2)		1.2 (2.6)		1.4 (3.0)		1.4 (2.3)		0.9 (1.4)		0.6 (1.2)		1.1 (2.1)		3.3 (4.7)	
	Male	Yes	2	4.5	0.328	1.0	0.370	1.3	0.769	3.9	0.476	1.1	0.509	0.7	0.670	1.0	0.729	4.0	0.265
		No	84	6.7 (4.6)		1.9 (3.6)		1.1 (2.4)		2.0 (3.1)		1.3 (1.8)		0.9 (1.4)		1.1 (2.4)		5.0 (5.0)	
Metabolic syndrome[a]	Female	Yes	4	4.3 (3.5)	0.662	0.2 (0.9)	**0.008***	2.3 (5.1)	0.884	3.7 (7.4)	0.424	0.3 (1.7)	0.125	0.3 (1.5)	0.437	0.7 (5.3)	0.955	2.3 (13.4)	0.493
		No	72	4.7 (4.4)		1.3 (2.7)		1.4 (2.8)		1.6 (2.3)		0.9 (1.5)		0.6 (1.2)		1.1 (2.3)		3.9 (4.7)	
	Male	Yes	3	6.8	0.877	5.5	0.316	2.3	0.152	4.0	0.402	0.5	0.560	0.7	0.929	0.8	0.515	1.9	0.083
		No	83	6.7 (4.6)		1.9 (3.2)		1.1 (2.3)		2.0 (3.1)		1.3 (1.6)		0.9 (1.4)		1.1 (2.4)		5.0 (5.0)	

*significant at $p<0.05$

N=305 (females) and N=318 (males), except for obesity (underweight subjects were excluded) and biomarkers (a subset of respondents underwent laboratory investigations).

IQR not provided for n<4

Supplementary Table S4: Median daily servings of food intake compared by metabolic risk factors according to ethnicity.

Metabolic risk factors	Ethnicity		n	Cereals & cereal products		Fruits		Vegetables		Meat, poultry & eggs		Fish and seafood		Legumes, seeds & nuts		Milk & dairy products		Processed foods & beverages	
				**Median (IQR)**	**P value**	**Median (IQR)**	**P value**	**Median (IQR)**	**P value**	**Median (IQR)**	**P value**	**Median (IQR)**	**P value**	**Median (IQR)**	**P value**	**Median (IQR)**	**P value**	**Median (IQR)**	**P value**
Obesity	Malay	Yes	237	6.5 (4.5)	0.248	2.1 (4.1)	0.577	1.2 (2.4)	0.546	1.9 (2.9)	0.788	1.3 (1.6)	0.822	0.7 (1.4)	0.948	0.9 (2.1)	0.599	4.2 (4.9)	0.141
		No	161	6.0 (4.6)		1.9 (3.2)		1.1 (3.0)		2.0 (3.3)		1.1 (1.7)		0.7 (1.3)		1.0 (1.7)		5.0 (5.2)	
	Chinese	Yes	42	6.2 (4.1)	0.329	3.1 (6.9)	**<0.001****	2.5 (3.5)	0.915	1.0 (1.8)	0.798	0.6 (1.5)	0.881	1.0 (1.6)	0.810	1.2 (2.8)	0.831	5.5 (5.5)	0.260
		No	33	6.3 (5.2)		1.4 (1.7)		2.8 (2.9)		1.0 (2.1)		0.6 (1.7)		0.7 (1.6)		1.4 (3.8)		4.6 (7.0)	
	Indian	Yes	69	3.9 (2.8)	0.756	1.8 (2.5)	0.203	1.2 (2.6)	0.765	2.0 (2.8)	0.778	0.4 (1.1)	0.906	0.7 (1.1)	**0.033***	0.6 (2.0)	0.797	2.9 (3.1)	0.757
		No	57	3.5 (3.9)		1.6 (2.1)		1.2 (2.4)		1.9 (2.0)		0.6 (1.1)		0.4 (0.8)		0.9 (1.5)		2.7 (3.9)	
Abdominal obesity	Malay	Yes	104	6.8 (5.0)	0.666	2.2 (4.8)	0.360	1.4 (3.1)	0.075	1.7 (3.1)	0.829	1.4 (1.7)	0.423	0.6 (1.2)	0.126	0.8 (1.7)	0.124	4.2 (5.0)	0.198
		No	312	6.0 (4.5)		1.9 (3.2)		1.1 (2.7)		2.0 (3.3)		1.1 (1.7)		0.8 (1.4)		1.0 (1.8)		5.0 (5.2)	
	Chinese	Yes	19	6.3 (2.5)	0.940	3.5 (8.4)	**0.014***	3.2 (3.5)	0.166	1.0 (1.0)	0.958	0.6 (1.2)	0.503	1.3 (1.6)	0.467	1.5 (3.8)	0.557	5.5 (5.0)	0.557
		No	59	6.2 (4.7)		1.9 (2.2)		3.1 (3.0)		1.0 (1.9)		0.6 (1.7)		0.7 (1.6)		1.2 (2.8)		5.0 (6.8)	
	Indian	Yes	28	4.0 (3.8)	0.884	1.7 (2.4)	0.734	1.2(2.4)	0.468	2.1 (2.5)	0.691	0.4 (1.4)	0.846	0.9 (1.1)	0.175	0.4 (2.6)	0.294	2.1 (2.2)	0.324
		No	101	3.7 (3.8)		1.6 (2.2)		1.2 (2.7)		1.9 (2.0)		0.5 (1.0)		0.5 (0.9)		0.9 (1.4)		2.9 (3.9)	
Elevated blood pressure	Malay	Yes	30	6.5 (5.84	0.434	1.8 (4.7)	0.720	1.0 (1.9)	0.621	1.4 (1.9)	0.300	1.0 (1.0)	0.142	0.6 (1.4)	0.577	0.6 (0.9)	0.081	3.8 (3.2)	**0.015***
		No	386	6.2 (4.5)		1.9 (3.3)		1.2 (2.8)		2.0 (3.3)		1.2 (1.7)		0.7 (1.4)		1.0 (1.9)		4.9 (5.3)	
	Chinese	Yes	3	9.1	0.170	2.3	0.750	0.8	0.146	1.0	0.519	0.3	**0.027***	1.3	0.864	1.3	0.903	4.6	0.981
		No	75	6.2 (4.2)		2.0(2.7)		2.6 (3.1)		1.0 (1.9)		0.6 (1.6)		0.7 (1.6)		1.2 (3.6)		5.2 (6.5)	
	Indian	Yes	8	4.0 (4.6)	0.747	1.2 (1.0)	0.151	2.0 (2.5)	0.945	5.5 (5.4)	**0.003***	0.5 (0.9)	0.488	0.8 (1.3)	0.404	0.3 (0.4)	**0.015***	1.5 (0.8)	**0.026***
		No	121	3.7 (3.6)		1.8 (2.3)		1.2 (2.5)		1.9 (2.0)		0.5 (1.1)		0.6 (1.0)		0.9 (1.8)		3.0 (3.7)	
Elevated triglyceride[a]	Malay	Yes	10	8.1 (4.6)	0.242	1.6 (5.3)	0.625	1.4 (3.2)	0.573	1.9 (3.5)	0.830	1.0 (2.0)	0.455	0.4 (1.1)	0.330	0.9 (1.2)	0.591	4.8 (5.3)	0.694
		No	120	6.3 (4.6)		1.8 (3.1)		1.1 (2.8)		2.0 (3.0)		1.2 (1.8)		0.8 (1.4)		1.4 (2.2)		5.0 (5.0)	
	Indian	Yes	3	2.4	0.393	0.7	0.057	0.6	0.437	1.0	0.856	0.2	0.139	0.7	0.914	0.8	0.393	1.37	0.162
		No	24	3.4 (1.3)		1.2 (1.5)		2.0 (2.6)		2.1 (2.4)		0.5 (1.1)		0.7 (1.1)		0.5 (0.7)		2.0 (2.2)	
Low HDL cholesterol[a]	Malay	Yes	10	6.8 (5.3)	0.544	2.2 (4.7)	0.868	1.5 (3.3)	0.834	3.6 (2.9)	0.099	2.3 (3.4)	0.055	1.8 (2.0)	0.468	1.5 (3.7)	0.133	6.2 (6.2)	0.861
		No	120	6.3 (4.6)		1.6 (3.1)		1.1 (2.7)		1.8 (2.9)		1.1 (1.7)		0.8 (1.2)		1.1 (2.2)		4.9 (4.9)	
	Indian	Yes	3	2.9	0.673	1.2	0.391	1.0	0.391	6.1	**0.019***	0.2	0.351	0.7	0.622	0.5	0.524	1.6	0.622
		No	25	3.5 (1.5)		0.9(1.5)		2.3 (2.5)		2.0 (2.4)		0.4 (1.1)		0.7 (1.0)		0.4 (0.7)		1.6 (1.9)	
Elevated fasting blood glucose[a,b]	Malay	Yes	4	7.7 (3.0)	0.257	1.0 (3.2)	0.352	0.3 (4.2)	0.302	3.3 (4.4)	0.305	2.2 (6.5)	0.352	0.4 (0.6)	0.157	2.9 (5.8)	0.302	7.3 (10.8)	0.155
		No	126	6.3 (4.6)		1.9 (3.1)		1.2 (2.7)		2.0 (3.0)		1.1 (1.7)		0.8 (1.3)		1.1 (2.2)		4.9 (4.8)	

Metabolic syndrome[a]	Malay	Yes	5	6.8 (3.8)	0.889	0.3 (9.0)	0.361	4.0 (3.8)	0.087	1.4 (4.4)	0.932	0.5(5.0)	0.407	0.5 (1.9)	0.781	0.9 (3.6)	0.870	3.0 (10.1)	0.248
		No	125	6.3 (4.6)		1.9 (3.1)		1.1 (2.7)		2.0 (3.0)		1.2 (1.7)		0.8 (1.3)		1.3 (2.2)		5.0 (5.0)	
	Indian	Yes	2	2.6	0.222	1.0	1.000	1.2	0.476	7.4	**0.011***	0.2	0.132	0.5	0.640	0.4	0.894	1.5	0.640
		No	26	3.5 (1.8)		0.9 (1.5)		2.0 (2.5)		2.0 (2.3)		0.5 (1.1)		0.5 (1.1)		0.5 (0.7)		1.8 (2.1)	

*significant at $p<0.05$

N= 416 (Malays), N= 78 (Chinese) and N=129 (Indians), except for obesity (underweight subjects were excluded) and biomarkers (a subset of respondents underwent laboratory investigations).

[a]No Chinese respondents with elevated triglyceride, low HDL cholesterol, elevated fasting blood glucose, metabolic syndrome

[b]Indian respondents excluded as lack of number of respondents with elevated fasting blood glucose

IQR not provided for n<4

Supplementary Table S5: Anthropometry and biomarkers compared according to adherence to dietary guidelines.

	Cereals & cereal products			Fruits			Vegetables			Meat, poultry & eggs			Fish and seafood			Legumes, seeds & nuts			Milk & dairy products		
	**Adhered**	**Did not adhere**	**P value**	**Adhered**	**Did not adhere**	**P value**	**Adhered**	**Did not adhere**	**P value**	**Adhered**	**Did not adhere**	**P value**	**Adhered**	**Did not adhere**	**P value**	**Adhered**	**Did not adhere**	**P value**	**Adhered**	**Did not adhere**	**P value**
	**Median (IQR)**	**Median (IQR)**		**Median (IQR)**	**Median (IQR)**		**Median (IQR)**	**Median (IQR)**		**Median (IQR)**	**Median (IQR)**		**Median (IQR)**	**Median (IQR)**		**Median (IQR)**	**Median (IQR)**		**Median (IQR)**	**Median (IQR)**	
**BMI z-score**	0.3 (2.6)	0.6 (2.4)	0.470	0.8 (2.6)	0.4 (3.4)	0.053	0.4 (2.4)	0.7 (2.4)	0.547	0.7 (2.4)	0.5 (2.6)	0.453	0.7 (2.4)	0.4 (2.6)	0.181	0.7 (2.6)	0.5 (2.5)	0.196	0.9 (2.6)	0.4 (2.5)	**0.028***
**Waist circumference (cm)**	67.5 (17.6)	70.0 (19.0)	**0.030***	70.6 (19.5)	67.5 (18.4)	0.065	67.0 (18.3)	70.0 (19.6)	0.584	69.8 (18.4)	69.0 (19.5)	0.717	69.0 (20.6)	69.0 (18.6)	0.737	69.0 (20.0)	69.3 (19.0)	0.774	70.5 (17.3)	69.0 (19.4)	0.891
**Total cholesterol (mmol/L)**	4.5 (1.0)	4.7 (1.1)	0.978	4.5 (1.0)	4.7 (1.1)	0.262	4.7 (1.4)	4.5 (1.1)	0.650	4.6 (1.0)	4.6 (1.2)	0.754	47 (1.2)	4.5 (1.0)	0.111	4.5 (1.1)	4.6 (1.1)	0.110	4.5 (1.0)	4.6 (1.1)	0.684
**Triglyceride (mmol/L)**	0.9 (0.6)	0.9 (0.5)	0.257	0.9 (0.6)	0.9 (0.5)	0.372	0.9 (0.6)	0.9 (0.5)	0.766	0.9 (0.5)	0.9 (0.5)	0.560	0.9 (0.6)	0.9 (0.3)	0.322	0.9 (0.7)	0.9 (0.4)	0.618	1.0 (0.4)	0.9 (0.5)	0.499
**HDL-C (mmol/L)**	1.4 (0.4)	1.4 (0.4)	0.736	1.4 (0.4)	1.4 (0.5)	0.312	1.4 (0.5)	1.4 (0.4)	0.683	1.4 (0.4)	1.4 (0.3)	1.000	1.4 (0.4)	1.4 (0.4)	0.564	1.4 (0.3)	1.4 (0.5)	0.854	1.3 (0.3)	1.4 (0.4)	0.330
**LDL-C (mmol/L)**	2.6 (0.8)	28 (1.0)	0.673	2.8 (1.0)	2.8 (0.9)	0.468	2.7 (1.3)	2.7 (0.9)	0.592	2.7 (0.8)	2.8 (1.1)	0.785	2.8 (1.0)	2.7 (1.0)	0.325	2.6 (1.1)	2.8 (0.9)	0.073	2.5 (0.8)	2.8 (1.0)	0.720
**Total cholesterol: HDL-C ratio**	3.3 (0.9)	3.5 (1.3)	0.903	3.4 (0.9)	3.4 (1.3)	0.789	3.7 (1.3)	3.4 (1.0)	0.319	3.3 (1.1)	3.4 (1.1)	0.616	3.3 (1.3)	3.4 (1.0)	0.826	3.2 (1.1)	3.5 (1.1)	0.216	3.3 (1.0)	3.4 (1.2)	0.735
**Fasting plasma glucose (mmol/L)**	4.6 (0.7)	4.7 (0.6)	0.244	4.7 (0.5)	4.7 (0.7)	0.452	4.9 (0.8)	4.7 (0.6)	0.254	4.8 (0.7)	4.7 (0.6)	0.182	4.7 (0.6)	4.7 (0.6)	0.526	4.7 (0.5)	4.8 (0.6)	0.544	4.7 (0.7)	4.7 (0.6)	0.314
**Systolic blood pressure (mmHg)**	110.0 (15.0)	110.0 (15.0)	0.177	109.0 (14.0)	110.0 (16.0)	0.251	111.0 (14.0)	111.0 (16.0)	0.885	109.0 (14.0)	111.0 (15.0)	0.072	107.0 (13.0)	111.0 (15.0)	**0.001***	110.0 (16.0)	110.0 (15.0)	0.721	110.0 (16.0)	109.0 (15.0)	0.748
**Diastolic blood pressure (mmHg)**	67.0 (14.0)	66.0 (13.0)	0.743	66.0 (13.0)	66.0 (13.0)	0.738	68.0 (15.0)	67.0 (12.0)	0.740	66.0 (13.0)	67.0 (14.0)	0.808	66.0 (13.0)	66.0 (13.0)	0.981	66.0 (13.0)	66.0 (13.0)	0.815	68.0 (15.0)	66.0 (13.0)	0.862

*significant at p<0.05
